# Partial Identification of the Average Causal Effect in Multiple Study Populations: The Challenge of Combining Mendelian Randomization Studies

**DOI:** 10.1097/EDE.0000000000001526

**Published:** 2022-08-05

**Authors:** Elizabeth W. Diemer, Luisa Zuccolo, Sonja A. Swanson

**Affiliations:** From the a Department of Child and Adolescent Psychiatry, Erasmus MC, Rotterdam, the Netherlands; b CAUSALab, Harvard T.H. Chan School of Public Health, Boston, MA; c Department of Epidemiology, Harvard T.H. Chan School of Public Health, Boston, MA; d MRC Integrative Epidemiology Unit at the University of Bristol, Bristol, United Kingdom; e Department of Population Health Sciences, Bristol Medical School, University of Bristol, Bristol, United Kingdom; f Center for Health Data Science, Human Technopole Foundation, Milan, Italy; g Department of Epidemiology, Erasmus MC, Rotterdam, the Netherlands; h Department of Epidemiology, School of Public Health, University of Pittsburgh, Pittsburgh, PA.

**Keywords:** Instrumental variable, Mendelian randomization, Partial identification, Research synthesis

## Abstract

Researchers often use random-effects or fixed-effects meta-analysis to combine findings from multiple study populations. However, the causal interpretation of these models is not always clear, and they do not easily translate to settings where bounds, rather than point estimates, are computed.

**Methods:**

If bounds on an average causal effect of interest in a well-defined population are computed in multiple study populations under specified identifiability assumptions, then under those assumptions the average causal effect would lie within all study-specific bounds and thus the intersection of the study-specific bounds. We demonstrate this by pooling bounds on the average causal effect of prenatal alcohol exposure on attention deficit-hyperactivity disorder symptoms, computed in two European cohorts and under multiple sets of assumptions in Mendelian randomization (MR) analyses.

**Results:**

For all assumption sets considered, pooled bounds were wide and did not identify the direction of effect. The narrowest pooled bound computed implied the risk difference was between −4 and 34 percentage points.

**Conclusions:**

All pooled bounds computed in our application covered the null, illustrating how strongly point estimates from prior MR studies of this effect rely on within-study homogeneity assumptions. We discuss how the interpretation of both pooled bounds and point estimation in MR is complicated by possible heterogeneity of effects across populations.

When data from multiple study populations are available, combining evidence across populations can improve our understanding of causal effects. For example, researchers commonly attempt to synthesize information from multiple studies using meta-analysis, which can improve precision by combining study-specific point estimates using either random-effects or fixed-effects models to obtain pooled effect estimates.^1–3^ However, the causal interpretation of estimates derived from these meta-analyses is not always clear, especially when random-effects models are used.^4,5^ Moreover, traditional meta-analytic approaches do not readily translate to pooling information from studies in which bounds rather than point estimates are computed.^6,7^

Here, we describe and apply an alternative approach to standard meta-analysis, which pools information from study-specific bounds as opposed to study-specific point estimates. In brief, we demonstrate how, if each study is viewed as a random sample from the same well-defined superpopulation, logical combinations of the data and underlying assumptions allow for partial identification of causal effects by the intersection or union of the bounds computed in each study.^5^ Although bounds can be computed in a variety of study designs, our application focuses on pooling two sets of Mendelian randomization (MR) analyses, an application of instrumental variable methods proposing genetic variants as instruments, to bound the average causal effect of alcohol consumption during pregnancy on offspring attention deficit-hyperactivity disorder (ADHD) symptoms.^8^ The individual studies computed bounds under many different sets of assumptions, as they had proposed multiple genetic variants as instruments,^8,9^ thereby giving an opportunity to explore how different sets of assumptions may come together in this pooled approach. We begin by describing the general theory.

## POOLING k BOUNDS COMPUTED ACROSS k STUDIES

Suppose we are interested in the average causal effect, also known as the causal risk difference, of an exposure *A* on an outcome *Y*, $E\left(Y^{a}-Y^{a^{\prime }}\right)$, in some well-defined population (with superscripts denoting counterfactuals, here setting levels of the exposure to values *a* and *a*′). We conduct *k* studies, which we will index with *S* = {1, 2, … *k*}, and within each study have computed bounds [LB_s_, UB_s_] on this population average causal effect for each of the *k* studies under some arbitrary set of identifiability assumptions. Then, assuming all sets of identifiability assumptions hold, the average causal effect $E\left(Y^{a}-Y^{a^{\prime }}\right)$ is bounded by the intersection of all these bounds, that is, $[\underset{s}{max}\;\left(LB_{s}\right),\underset{s}{min}\;(UB_{s})].$ A simple proof of this is given in the eAppendix; http://links.lww.com/EDE/B950. (We note that we are not claiming that these bounds are sharp; see eAppendix; http://links.lww.com/EDE/B950.) Notably, if the intersection of the bounds computed in each study is an empty set, that is evidence that at least one of the identifiability assumptions in at least one study is violated.

Before continuing, we wish to flag that the logic of the above statements, and the proof in the eAppendix; http://links.lww.com/EDE/B950, rely on several subtle points that merit scrutiny in practice. In particular, the interpretation of the bounds computed in each study as bounds on the population average causal effect will rely on principles that have been described in the context of transportability.^4^ Namely, we must have a well-defined target population in mind, that is, a group of individuals that the investigators wish to conduct inference on, whose group boundaries are sufficiently clearly specified based on subject matter knowledge. Further, we must specify why each of these studies are targeting an effect in that population. Most often, this will require some form of homogeneity assumption,^10^ as implicitly is required for interpretability of traditional fixed-effect meta-analyses. In this case, we assume that the exposure–outcome effect does not differ by study population on the relevant scale (here, additive). This method also assumes consistency of counterfactual outcomes across the target population and included study populations (meaning that if $A_{i}=a$, then $Y_{i}^{a}=Y_{i}$ for every individual i). We return to these challenges, as well as issues of sampling variability, in the discussion.

Notably, set intersections are not the only means of combining information from separate studies’ bounds. Suppose, for example, we wished to compute bounds under the assumption that at least one of the *k* studies’ identifiability assumptions held, but we do not have evidence of which study. In that case, the average causal effect would lie in the union of the bounds from each study population, that is, $\left[\underset{s}{min}\;\left(LB_{s}\right),\underset{s}{max}\;\left(UB_{s}\right)\right]$.^9^ However, in many settings, it is difficult to imagine a bias that would invalidate at least one study without invalidating all included studies, particularly if the same identifiability assumptions are evoked for computing all study-specific bounds. One example may be if studies had different types or amounts of attrition, and different ways of mitigating such selection bias due to attrition that were study-specific.

## POOLING MULTIPLE (>k) BOUNDS COMPUTED ACROSS k STUDIES UNDER MULTIPLE SETS OF ASSUMPTIONS

A single study may present multiple opportunities to bound the same average causal effect under slightly different identifiability assumptions. For example, in Mendelian randomization (MR), researchers often propose multiple genetic variants as instruments to estimate the same exposure–outcome relation. In this case, researchers could consider generating bounds separately for each genetic variant, under the assumption that each genetic variant was a valid instrument on its own. Alternatively, under the assumption that multiple genetic variants were individually and jointly valid instruments, bounds could be calculated by proposing a set of genetic variants as a joint instrument.^8^ This approach could be applied not only to the complete set of genetic variants proposed as instruments, but also to every possible subset of those genetic variants (in a similar spirit to a “leave-one-out” meta-analysis approach). Note that, when using methods like inverse-probability weighting or standardization to account for measured proposed instrument-outcome confounders, investigators must only assume that the genetic variant(s) are conditionally valid instruments, rather than marginally valid instruments.

In this case, bounds can be pooled across study populations separately for each assumption set used to generate the bounds. In an MR study proposing multiple genetic variants as instruments, investigators can generate pooled bounds on $E\left(Y^{a}-Y^{a^{\prime }}\right)$ separately for each subset of genetic variants proposed as instruments. These pooled bounds can then be compared with one another to “triangulate” results and, by assessing the degree of overlap between different pooled bounds, to evaluate the dependence of the results on the validity of the MR conditions for each genetic variant proposed as an instrument.

In addition, investigators can consider pooling bounds across different sets of assumptions, such as pooling MR bounds with variations on the assumption-free bounds that do not require MR conditions.^11^ If two studies computed bounds on the same average causal effect using methods that relied on two different assumption sets, then we would expect the average causal effect to be within the intersection of those bounds under the combined (but study-specific) assumptions.

### Application

#### Data

We computed pooled bounds on the average causal effect of maternal alcohol consumption during pregnancy on offspring ADHD, based on summary results of our previous MR analysis conducted in the Avon Longitudinal Study of Parents and Children (ALSPAC) and the Norwegian Mother, Father, and Child Study (MoBa).^8^ Further details on these cohorts are available in the eAppendix; http://links.lww.com/EDE/B950 and elsewhere.^12–16^ The only use of MoBa and ALSPAC data within the current study were secondary analysis of summary results from the aforementioned paper.^8^ Informed consent for the use of ALSPAC data collected via questionnaires and clinics was obtained from participants following the recommendations of the ALSPAC Ethics and Law Committee at the time. Ethical approval was obtained from the ALSPAC Ethics and Law Committee and the Local Research Ethics Committees. The establishment of MoBa and initial data collection was based on a license from the Norwegian Data Protection Agency and approval from The Regional Committee for Medical and Health Research Ethics. MoBa is now based on regulations related to the Norwegian Health Registry Act. For any given single nucleotide polymorphism (SNP) *Z*, partial identification of the average causal effect can be achieved if the following MR conditions hold: (1) *Z* is associated with the exposure *A*, (2) *Z* has no effect on the outcome *Y* except through the exposure *A*, and (3) individuals with different genotypes for *Z* are exchangeable with regards to counterfactual outcome (i.e., *Z* is not confounded with *Y* or related through selection bias).^17,18^ If *Z* satisfies all three assumptions, *Z* is considered a valid instrument for the effect of *A* on *Y*. When a set of SNPs are proposed as joint instruments, all three conditions must hold for the set of SNPs individually as well as jointly. Importantly, conditions 2 and 3 are not verifiable (thought they can at times be falsified).^17,19^ The previous study laid out several reasons why the MR conditions may not hold in this context, including selection on pregnancy, various forms of pleiotropy, assortative mating, and time-varying SNP-exposure relationships.^8^

We previously computed bounds under the MR model in each cohort separately, proposing 11 maternal SNPs (rs145452708, rs193099203, rs11940694, rs29001570, rs3114045, rs140280172, rs9841829, rs35081954, rs9991733, rs149127347, chr18:72124965) as instruments for the effect of any alcohol consumption during pregnancy on offspring ADHD. Within MoBa, rs145441283 was used as a proxy for rs193099203 and rs1154447 was used as a proxy for rs35081954. Because chr18:72124965 was unavailable in either cohort, rs201288331 was used as proxy in ALSPAC, and rs12955142 was used as a proxy in MoBa. The outcome was mother-reported ADHD symptoms in the clinical range in the offspring, measured using either the Development and Wellbeing Assessment or the Child Behavior Checklist Attention Deficit Hyperactivity subscale.^20,21^ In the first model, the exposure *A* = 1 if mothers reported any alcohol consumption during the second and third trimester of pregnancy, and *A* = 0 if they did not report any alcohol consumption. In the second model, mothers who consumed more than 32 g of alcohol per week (equivalent to approximately two cans of beer or glasses of wine) were removed from the analytic dataset. Although this second question focuses more explicitly on the effects of light alcohol consumption on offspring ADHD, conditioning on the exposure in this way can result in selection bias.^22^

#### Statistical Analyses Conducted in the Prior Study

Analyses in both cohorts were restricted to mother–child pairs without missing data on the exposure, outcome, or any of the proposed genetic instruments. Within ALSPAC, analyses were restricted to participants of self-reported white British ancestry. Because MoBa does not collect data on self-reported ancestry, we did not restrict the MoBa sample based on ancestry. However, only 5.6% of all MoBa participants report a first language other than Norwegian, suggesting the study population is primarily of Scandinavian ancestry.^23^ These restrictions resulted in analytic samples of 2,056 mother–child pairs in ALSPAC and 6,216 mother–child pairs in MoBa. Prevalence of alcohol consumption and ADHD symptoms in both cohorts are shown in Table 1. To limit the impact of residual population stratification, we estimated inverse probability weights for 10 principal components (see eAppendix; http://links.lww.com/EDE/B950). All analyses were then conducted in the inverse-probability weighted pseudopopulation. However, it should be noted that this approach may not fully capture variation in genetic ancestry, particularly if cryptic relatedness is present within the sample.

**TABLE 1.** Prevalence of Maternal Alcohol Use and Offspring Attention Deficit-Hyperactivity Disorder Symptoms in the ALSPAC and the Norwegian Mother, Father, and Child Study (MoBa)

	ALSPAC (n = 2,056)	MoBa (n = 6,216)
	% (n)	% (n)
Alcohol use during second and third trimester of pregnancy		
0 g/week	45.1 (927)	90.1 (5,603)
>0–32 g/week	23.1 (474)	9.0 (562)
>32 g/week	31.9 (655)	0.8 (51)
Offspring attention deficit-hyperactivity disorder symptoms	2.3 (47)	2.6 (163)

Before calculating bounds, we had eliminated combinations of SNPs proposed as instruments for which the MR conditions were falsified.^19,24,25^ For each set that was not falsified, bounds were then calculated using the methods described by Richardson and Robins^26^ (see eSupplementary Materials; http://links.lww.com/EDE/B950, for further details).

#### Statistical Analysis for the Pooled Results

To pool results, we assumed the bounds computed within ALSPAC and MoBa identify the average causal effect in the population of western European mother–child pairs. Here, we assumed that any assumption set that was falsified in either cohort represented a structural violation of the MR conditions in the population of interest and removed the set from further analysis.

Otherwise, for each subset of the SNPs proposed as instruments, we pooled bounds by taking the intersection of bounds calculated in ALSPAC and MoBa. Although it is possible that cohort-specific selection biases were present in only one cohort, or that control for residual population stratification was insufficient in only one of the cohorts, we do not have a strong a priori reason to believe that a source of bias might exist that is completely unique to only one cohort. Therefore, we do not present union bounds.

All analyses were conducted in R version 3.6.1 (R Foundation for Statistical Computing, Vienna, Austria).

## RESULTS

We first consider pooling bounds on the effect of any alcohol consumption compared with no alcohol consumption during pregnancy among European women, proposing single SNPs as instruments. As shown in Table 2, under the assumptions that the SNP in question is a valid instrument in both study populations, and that there is no effect modification by study population, the estimated bounds on the average causal effect will be the intersection of the bounds calculated in each cohort. For example, when rs11940694 is proposed as an instrument, bounds implied the risk difference was between −51 and 43 percentage points in ALSPAC, −11 and 88 percentage points in MoBa, and therefore the pooled bounds imply a risk difference between −11 and 43 percentage points. Notably, because the instrumental inequalities failed to hold for four individual SNPs in MoBa, we have evidence that those SNPs are not valid instruments in at least one cohort (MoBa), and therefore do not meet the assumptions we necessitate for pooling. For every SNP proposed as an instrument individually, the pooled bounds were wide and consistent with maternal alcohol consumption slightly decreasing risk of offspring ADHD, having no effect, or increasing risk of offspring ADHD.

**TABLE 2.** Bounds on the Average Causal Effect of Any Alcohol Consumption During Pregnancy on Offspring Attention Deficit-hyperactivity Disorder Symptoms in Each Cohort and Pooled Across Cohorts, Assuming Single Genetic Variants Are Individually Valid Instruments

	ALSPAC		MoBa		Pooled		
Proposed Instrument	Lower Bound	Upper Bound	Lower Bound	Upper Bound	Lower Bound	Upper Bound	Key Assumptions for Pooled Bounds
rs11940694	−0.51	0.43	−0.11	0.88	−0.11	0.43	1. rs11940694 is a valid instrument in both studies 2. No effect modification by study population 3. Consistency
rs140280172	−0.47	0.46	NA	NA	NA	NA	
rs145452708	−0.47	0.47	−0.03	0.87	−0.03	0.47	1. rs145452708 is a valid instrument in both studies 2. No effect modification by study population 3. Consistency
rs149127347	−0.45	0.47	NA	NA	NA	NA	
rs193099203	−0.52	0.32	NA	NA	NA	NA	
rs201288331	−0.52	0.17	NA	NA	NA	NA	
rs29001570	−0.39	0.46	−0.10	0.87	−0.10	0.46	1. rs29001570 is a valid instrument in both studies 2. No effect modification by study population 3. Consistency
rs3114045	−0.47	0.45	−0.08	0.88	−0.08	0.45	1. rs3114045 is a valid instrument in both studies 2. No effect modification by study population 3. Consistency
rs35081954	−0.52	0.45	−0.10	0.88	−0.10	0.45	1. rs35081954 is a valid instrument in both studies 2. No effect modification by study population 3. Consistency
rs9841829	−0.52	0.41	−0.07	0.87	−0.07	0.41	1. rs9841829 is a valid instrument in both studies 2. No effect modification by study population 3. Consistency
rs9991733	−0.52	0.40	−0.10	0.88	−0.10	0.40	1. rs9991733 is a valid instrument in both studies 2. No effect modification by study population 3. Consistency

Bounds are noted as NA when the Mendelian randomization conditions were falsified within a cohort, and the assumptions for pooling were not met. Within this table, the statement that a SNP is a valid instrument indicates that the SNP is a valid instrument conditional on 10 principal components, not necessarily that the SNP is a marginally valid instrument.

When pooling the bounds computed in each cohort assuming multiple SNPs were valid instruments, the pooled bounds are slightly narrower than those generated proposing individual SNPs as instruments (Table 3), with the narrowest pooled bound computed implying the risk difference was between −4 and 34 percentage points. Overall, similar to bounds generated proposing single SNPs as instruments, the pooled bounds were consistent with maternal alcohol consumption slightly reducing risk of offspring ADHD, having no effect, or increasing risk of offspring ADHD. The pooled bounds were generally similar when computing bounds for the effect of light alcohol consumption (Figure).

**TABLE 3.** Pooled Bounds on the Average Causal Effect of Any Alcohol Consumption During Pregnancy on Offspring Attention Deficit-hyperactivity Disorder Symptoms in Each Cohort and Pooled Across Cohorts, Assuming Multiple Genetic Variants Are Individually and Jointly Valid Instruments

	ALSPAC		MoBa		Pooled		
Proposed Joint Instruments	Lower Bound	Upper Bound	Lower Bound	Upper Bound	Lower Bound	Upper Bound	Key Assumptions for Pooled Bounds
{rs9991733, rs9841829}	−0.47	0.34	−0.04	0.84	−0.04	0.34	1. rs9991733 and rs9841829 are valid instruments in both study populations 2. No effect modification by study population 3. Consistency
{rs9991733, rs350819544}	−0.50	0.33	−0.08	0.86	−0.08	0.33	1. rs9991733 and rs35081954 are valid instruments in both study populations 2. No effect modification by study population 3. Consistency
{rs35081954, rs9841829}	−0.48	0.33	−0.06	0.84	−0.06	0.33	1. rs35081954 and rs9841829 are valid instruments in both study populations 2. No effect modification by study population 3. Consistency
{rs145452708, rs29001570}	−0.25	0.46	−0.08	0.87	−0.08	0.46	1. rs145452708 and rs29001570 are valid instruments in both study populations 2. No effect modification by study population 3. Consistency
{rs11940694, rs9841829}	−0.49	0.33	−0.06	0.84	−0.06	0.33	1. rs11940694 and rs9841829 are valid instruments in both study populations 2. No effect modification by study population 3. Consistency
{rs11940694, rs35081954}	−0.46	0.39	−0.07	0.85	−0.07	0.39	1. rs11940694 and rs35081954 are valid instruments in both study populations 2. No effect modification by study population 3. Consistency
{rs11940694, rs3114045}	−0.15	0.41	−0.07	0.84	−0.07	0.41	1. rs11940694 and rs3114045 are valid instruments in both study populations 2. No effect modification by study population 3. Consistency

Note: Within this table, the statement that a set of SNPs are valid instruments indicates that the SNPs are valid instruments conditional on 10 principal components for genetic ancestry, not necessarily that the set are marginally valid instruments.

**FIGURE.**
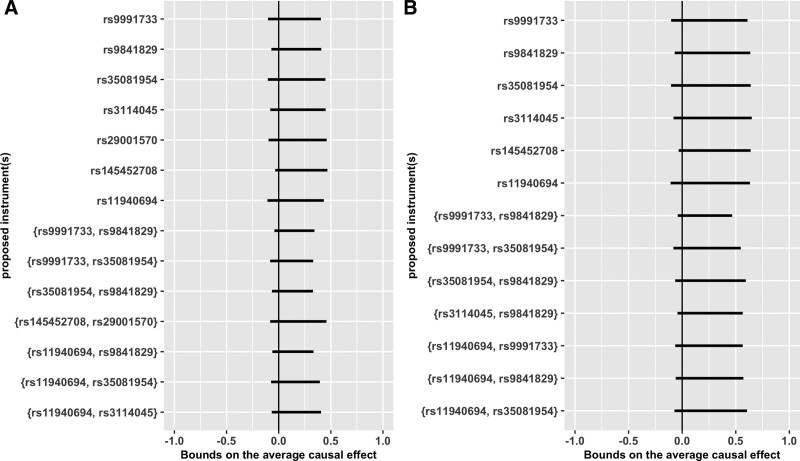
Pooled bounds on the average causal effect of alcohol consumption during pregnancy on offspring attention deficit-hyperactivity disorder symptoms under different exposure definitions, with inverse probability weighting to account for residual confounding. A shows bounds on the average causal effect of any alcohol consumption compared with no alcohol consumption during pregnancy, in a pseudo-population inverse probability weighted for 10 principal components. B shows bounds on the average causal effect of light alcohol consumption (≤32 g/week) in a pseudo-population inverse probability weighted for 10 principal components.

## DISCUSSION

Methods for combining bounds generated using MR in different studies have not been clearly established. Here, we demonstrate a straightforward approach for pooling MR bounds calculated in different cohorts with available individual-level data and clarify the assumptions necessary to perform such an analysis. Not only does this pooling procedure provide a method for synthesizing results from MR bounds analyses in multiple cohorts, it also will necessarily produce bounds that are equal in length or narrower than bounds computed in each cohort separately. In fact, because the narrowness of a set intersection bound depends both on the size of the bounds being pooled and their position relative to one another, pooling theoretically can yield substantially narrower bounds even when the bounds from each study population are fairly wide.^5^

As with any causal inference, it is critical to clearly define the population of interest. The ambiguity that results from an ill-defined population is compounded when we consider pooling data across studies.^4,27^ It is common to imagine study populations as being drawn from an infinite super-population of individuals meeting particular eligibility criteria, and to aim to extend inferences to that infinite super-population. For the current application, one could argue that we are interested in the effect of alcohol consumption during pregnancy on offspring ADHD among all women of western European ancestry living in western Europe who have become pregnant since the beginning of study recruitment, or will ever become pregnant in the future, a population that is effectively infinite. However, the idea that study populations were randomly sampled from such an infinite super-population is a fiction.^18,28^ Within our application, each study population was restricted to a particular country, and, like all studies, were restricted to particular time periods. Beyond this, previous research has found that participants in cohort studies differ from nonparticipants in meaningful ways.^29–32^

How then, are we able to justify using these study-specific data and results to bound a population average causal effect? One answer is that these pooling methods rely on an assumption of no effect modification of the exposure–outcome relation by study *S* on the relevant scale (here, additive). In practice, even if each study population was not a true random sample of the super-population, this assumption would hold if *S* was not related to either the outcome or any effect measure modifiers of the exposure–outcome relation. However, if the distribution of modifiers differed in different cohorts, this assumption would be violated. This is because the average causal effect in the super-population would be a weighted average of the effect within strata of the modifiers, with weights based on the distribution of modifiers in the super-population. Meanwhile, the pooled bounds we computed are based on the distribution of modifiers present within each cohort. If study populations differed in the distribution of an effect modifier from the super-population, then the average causal effect would not necessarily lie within the intersection or union or study-specific bounds.

Unfortunately, this homogeneity assumption is implausible in many settings, including ours. Where individual-level data on the proposed instrument, exposure, outcome, and potential effect modifiers are available in both the populations used to generate the bounds, and the distribution of potential effect modifiers is known for the target population of interest, it is possible that existing methods for transporting point estimates of average causal effects could be adapted to bounds of average causal effects to ameliorate this issue, that is, by reweighting or standardizing study populations to reflect the distribution of effect modifiers in a target population.^4,7^ In the specific context of MR, this is substantially more complicated in practice compared with alternative analyses, as many plausible effect modifiers may be downstream of the SNP proposed as an instrument. In the case of our application, for example, the effect of alcohol consumption in pregnancy on offspring ADHD may be modified by the speed at which a woman metabolizes alcohol, or by offspring genotype. Moreover, since many of the existing transportability methods require an assumption of homogeneity conditional on covariates,^4,10^ this may be further difficult to justify in the context of bounds computed under MR or other instrumental variable (IV)–based assumptions. Although the assumption of homogeneity conditional on known effect modifiers is certainly a weaker and more believable assumption, it is always possible that causal effects may still vary across populations as a result of differences in the distribution of unknown or unmeasured effect modifiers.^18^ An intrinsic motivation for the use of partial identification over point estimation in IV approaches is the desire to avoid strong, potentially implausible assumptions about the homogeneity of effects within study populations (most often the assumption that the exposure–outcome relation does not vary across levels of the proposed instrument).^7^ It is therefore somewhat troublesome that, to pool bounds across study populations, we must assume that, although the effect of interest may vary within a study population, the average causal effect is homogenous across different study populations.

Although this issue presents a specific complication to the use of pooled bounds, it also highlights a broader issue with the conduct and interpretation of MR studies, which are now frequently being used as evidence for policy interventions.^33–35^ This includes the application here, in which previous MR studies on alcohol consumption during pregnancy have been cited in support of policy recommendations.^36,37^ Yet, in both MR studies and other designs, the study populations effects are estimated in are not necessarily selected randomly from the population in which the guidelines or policies are being given. This is all the more true when MR study populations are restricted to white European ancestry groups to avoid bias from population stratification.^38^ Extending inferences from these MR studies to a defined population then also requires homogeneity assumptions. Regardless of whether such a homogeneity assumption might truly hold, they are rarely, if ever, discussed. As has been highlighted in previous research, further study is needed to increase the availability of genetic data in diverse populations.^39^ However, for MR studies, it will also be critical for future research to carefully consider how causal effects might vary across populations of interest, or across potentially relevant variables.

There is also an issue of consistency in exposure definition that one needs to consider: pooling study-specific bounds on a population average causal effect is further complicated in practice by whether consistency can be reasonably assumed. Formally, these pooling methods require that if $A_{i}=a$, then $Y_{i}^{a}=Y_{i}$ for every individual i in the target population and the included study populations. This implies that the exposure of interest must be the same across studies, and that study participation does not impact the outcome. Similar to the issues of heterogeneity presented above, this issue points to a broader issue with the interpretation and comparison of findings across multiple designs, including MR and randomized trials, where the duration and dose of exposure might vary substantially. For example, within observational studies, the assumption of consistency across studies may become especially problematic when a single binary exposure encompasses several versions of treatment, but the distribution of those treatment versions differs between study populations. In our primary example, we have grouped into two categories, never versus ever drinking during pregnancy. However, beyond possible issues of measurement error, it is likely that the amount of drinking, and not just the presence, during pregnancy affects ADHD symptom risk. If so, and individuals in each study population who consume alcohol differ in the amount of alcohol they consume, then there is relevant treatment variation,^27^ and the causal interpretation of bounds pooled across these study populations would be unclear. This may be an especially important consideration when evaluating studies of nonpregnancy exposures, where the duration of exposure could vary substantially across study populations. Intuitively, it is easy to see why bounds pooled from an MR study targeting a “lifetime effect” and a randomized trial with a very short window of exposure would have no clear interpretation. The timing of exposure in these two cases differ dramatically, and if said timing has an impact on outcomes, a pooled bound from these two studies would not have a clear interpretation.^40^ However, it is important to recognize that this same issue would also affect comparisons between multiple MR studies of “lifetime effects.” In such MR designs, a single definition of the exposure could encompass many different exposure trajectories over the life-course, the distribution of which may differ between study populations.

We have focused primarily on identification, without discussing issues of statistical imprecision. However, bounds are impacted by the uncertainty created by sampling variation. Indeed, because the proofs presented in our eAppendix; http://links.lww.com/EDE/B950, assume that the sample-specific bounds accurately reflect the super-population bounds, and do not account for the impact of sampling variability, the intersection methods presented here may actually result in overly narrow bounds when applied to real data. However, it should be noted that, within our applied example, the pooled bounds were fairly wide for all combinations of proposed genetic instruments, suggesting the potentially optimistic nature of these bounds has limited impact to our application and perhaps other similar MR contexts. Nonetheless, clear strategies for incorporating sampling variability will be critical to the use of pooled bounds in practice. That this is an area of active methodologic developments is reflected in the fact that, despite a growing literature on confidence interval estimation and statistical inference for bounds,^6,7,41,42^ currently there is no consensus on the best approach to accounting for this uncertainty, including for the set intersection methods we describe. This is doubly important for MR studies that use the instrumental inequalities for falsification, as statistical inference for the instrumental inequalities themselves has some shared challenges with inference for bounds on the average causal effect.^43^ Estimation would also be further complicated if the population of interest was in fact finite.^44^

In our applied example, while bounds on the effects of prenatal alcohol exposure on ADHD did narrow, they did not identify a direction of effect. Readers might therefore question whether the many complications of pooling in practice are worthwhile, or how such pooled bounds could actually be integrated into decisionmaking. Importantly, bounds do not necessarily replace point identification strategies, and can be presented alongside point estimates. Indeed, to make recommendations about drinking behaviors and offspring ADHD risk based on these MR applications, we would need either to add further point-identifying assumptions or to use another causal inference approach.

Yet, integrating bounds alongside point estimates has a number of advantages that could benefit both the interpretation of causal effects from MR studies and their translation into policy and practice. As has been extensively argued previously, bounds, especially wide bounds, can help show how strongly a particular analysis relies on assumptions.^7,11,45–49^ Within individual MR studies with multiple SNPs proposed as instruments, computing bounds using different subsets of SNPs allows investigators to evaluate how results are affected by assumptions about both homogeneity and the validity of specific SNPs proposed as instruments, and even offer an alternative to so-called pleiotropy-robust methods. By quantifying the degree to which an analysis depends on such assumptions, bounding approaches can identify cases where potential violations of these assumptions should be more closely evaluated.

In the context of meta-analyses of MR results, pooled bounds are not directly comparable to fixed- or random-effects estimation, and therefore they should not be seen as an alternative but rather as a complementary strategy. Incorporating pooled bounds into meta-analyses provides an opportunity to show how the conclusions of such an analysis might be impacted by heterogeneity of effects within studies. The use of such bounds also highlights the implicit assumption of homogeneity of effects and consistency across populations made whenever MR estimates are generalized to broader populations. By making these assumptions explicit, pooling approaches could help researchers and readers to identify areas in need of further investigation (e.g., evaluation of the extent to which effects of interest vary across populations of interest).

## CONCLUSIONS

The use of pooled-bounding methods in practice is complicated by issues of effect homogeneity, definitions of populations of interest, and consistency. Although these issues pose a challenge to the use of pooling or meta-analytic methods, they also illuminate the implicit assumptions made each time MR estimates are used to inform policy recommendations or are being “triangulated” with other study results. The presentation of bounds across different assumption sets can help clarify the extent to which the conclusions of an analysis depend on the assumptions made.

## ACKNOWLEDGMENTS

The Norwegian Mother, Father and Child Cohort Study is supported by the Norwegian Ministry of Health and Care Services and the Ministry of Education and Research. We are grateful to all the participating families in Norway who take part in this ongoing cohort study. We thank the Norwegian Institute of Public Health (NIPH) for generating high-quality genomic data. This research is part of the HARVEST collaboration, supported by the Research Council of Norway (#229624). We also thank the NORMENT Centre for providing genotype data, funded by the Research Council of Norway (#223273), South East Norway Health Authorities and Stiftelsen Kristian Gerhard Jebsen. We further thank the Center for Diabetes Research, the University of Bergen for providing genotype data and performing quality control and imputation of the data funded by the ERC AdG project SELECTionPREDISPOSED, Stiftelsen Kristian Gerhard Jebsen, Trond Mohn Foundation, the Research Council of Norway, the Novo Nordisk Foundation, the University of Bergen, and the Western Norway Health Authorities. We are extremely grateful to all the families who took part in this study, the midwives for their help in recruiting them, and the whole ALSPAC team, which includes interviewers, computer and laboratory technicians, clerical workers, research scientists, volunteers, managers, receptionists, and nurses.

## Supplementary Material


