# Supplementary material for: Partial Identification of the Average Causal Effect in Multiple Study Populations: The Challenge of Combining Mendelian Randomization Studies
Source: Epidemiology. 2022 Aug 5;34(1):20–8. doi: 10.1097/EDE.0000000000001526 (PMC9719801; doi:10.1097/EDE.0000000000001526)
Supplement: Supplementary file 1 [file ede-34-020-s001.docx]

**Supplementary Materials**

**Table of Contents**

1. Pooling Bounds on causal parameters across multiple study populations

2. Impact of sampling variability on pooled bounds

3. Sharpness of Intersection Bounds

4. Description of the Avon Longitudinal Study of Parents and Children

5. Description of the Norwegian Mother, Father, and Child Study

6. Expression for the Richardson-Robins bounds

7. Inverse probability weighting for principal components for genetic ancestry

8. R code for generating and graphing intersection bounds

1. **Pooling Bounds on causal parameters across multiple study populations**

The logic of these proofs closely mirror those given by Swanson (Swanson 2017). We consider bounding the average causal effect of a treatment *A* on an outcome *Y* in some super population of interest, E$(Y^{a}-Y^{a^{'}})$, where superscripts denote counterfactuals. From the super-population, researchers have drawn a collection of study populations, $S=\{1,2,..,k\}$. For simplicity, let LB_s_ and UB_s_ represent bounds on E$\left( Y^{a}-Y^{a^{'}} \right|S=s)$ , the study population-specific average causal effect.

***Set Intersection Method***

*Theorem*: Suppose that the assumptions used to calculate bounds within each study population hold, that E$\left( Y^{a}-Y^{a^{'}} | S=s \right)=E\left( Y^{a}-Y^{a^{'}} \right|S=s')$, and that if $A_{i}=a$, then $Y_{i}^{a}=Y_{i}$ for every individual *i* in each study population and the super-population. Then $E\left( Y^{a}-Y^{a^{'}} \right)\in[\max_{s} \left( LB_{s} \right),\min_{s} \left( UB_{s} \right)]$.

*Proof*:

For each study population *S = s* , if the assumptions used to calculate the bounds hold, then the study population average causal effect $E\left( Y^{a}-Y^{a^{'}} \right|S=s)$ is contained in the study-specific bounds $[LB_{s}, UB_{s}]$.

If $E\left( Y^{a}-Y^{a^{'}} \right|S=s)=E\left( Y^{a}-Y^{a^{'}} \right|S=s')$ , then $E\left( Y^{a}-Y^{a^{'}} \right|S=s)=E(Y^{a}-Y^{a^{'}})$.

Then $E(Y^{a}-Y^{a^{'}})$ is contained within $\left[ LB_{s}, UB_{s} \right]$ , the bounds on the study population – specific average causal effect.

If this holds for all *k* study populations, then the average causal effect $E\left( Y^{a}-Y^{a^{'}} \right)$ is contained within all *k* sets of bounds:

$$E\left( Y^{a}-Y^{a^{'}} \right)\in\left[ LB_{1},UB_{1} \right]$$

$$E\left( Y^{a}-Y^{a^{'}} \right)\in[LB_{2},UB_{2}]$$

…

$$E\left( Y^{a}-Y^{a^{'}} \right)\in[LB_{k}, UB_{k}]$$

Taking the intersection of these sets, $E\left( Y^{a}-Y^{a^{'}} \right)$ is contained within $[\max_{k} \left( LB_{k} \right),\min_{k} \left( UB_{k} \right)]$.

***Set Union Method***

*Theorem*: Suppose that the assumptions used to calculate bounds within each study population hold in at least study population, that E$\left( Y^{a}-Y^{a^{'}} | S=s \right)=E\left( Y^{a}-Y^{a^{'}} \right|S=s')$, and that if $A_{i}=a$, then $Y_{i}^{a}=Y_{i}$ for every individual *i* in each study population and the super-population. Then $E\left( Y^{a}-Y^{a^{'}} \right)\in[\min_{s} \left( LB_{s} \right),\max_{s} \left( UB_{s} \right)]$.

*Proof*:

For an arbitrary study population *S = s* , if the assumptions used to calculate the bounds hold, then the study population average causal effect $E\left( Y^{a}-Y^{a^{'}} \right|S=s)$ is contained in the study-specific bounds $[LB_{s}, UB_{s}]$.

If $E\left( Y^{a}-Y^{a^{'}} \right|S=s)=E\left( Y^{a}-Y^{a^{'}} \right|S=s')$ , then $E\left( Y^{a}-Y^{a^{'}} \right|S=s)=E(Y^{a}-Y^{a^{'}})$.

Then $E(Y^{a}-Y^{a^{'}})$ is contained within $\left[ LB_{s}, UB_{s} \right]$ , the bounds on the study population – specific average causal effect.

Then, if the assumptions used to calculate the bounds hold in at least one study population s, then the population average causal effect is held within at least one of the *k* bounds. That is, if at least one of the following expressions are true:

$$E\left( Y^{a}-Y^{a^{'}} \right)\in[LB_{1},UB_{1}]$$

$$E\left( Y^{a}-Y^{a^{'}} \right)\in[LB_{2}, UB_{2}]$$

…

$$E\left( Y^{a}-Y^{a^{'}} \right)\in[LB_{k},UB_{k}]$$

Then the population average causal effect must be in the union of the above sets. Therefore, $E\left( Y^{a}-Y^{a^{'}} \right)\in[\min_{s} LB_{s},\max_{s} UB_{s}]$ .

Note: This means that the union bounds rely on less restrictive assumptions about the validity of the analyses within each study population than the intersection bounds. However, these union bounds still require the same homogeneity assumptions as the intersection bounds. It is difficult to imagine settings in which the source of the violation of the assumptions necessary to compute bounds within each study population does not also modify the average causal effect of interest. Further, when pooling bounds computed using the same assumption sets, the union bounds require that the source of violation be study-specific, and does not affect the validity of the model in the super-population (e.g., selection bias in MR studies proposing the same SNPs as instruments). Applications of the union bounds may therefore be limited to very specific contexts, such as studies of homogenous biological effects that may have been impacted by selection bias.

1. **Impact of sampling variability on the pooled bounds**

A key limitation of the proofs presented above is that they assume that the bounds calculated within each cohort accurately reflect the true super-population bounds, and are not impacted by sampling variability. However, in reality, the cohort-specific bounds will be impacted by sampling error, meaning that the methods presented here could underestimate the upper bounds and overestimate the lower bounds when applied to estimated bounds. As an illustrative example, one can consider the case where bounds on the average causal effect were estimated proposing the same SNP as an instrument in k random samples of the super-population of interest. Assuming the SNP was a truly valid instrument, the true super-population bounds would be exactly the same in each of the k cohorts. However, because of sampling variability, the estimated bounds from each of the k cohorts would differ from one another. It would then be very likely that the maximum value of the k cohort-specific lower bounds would overestimate the true lower bound in the super-population and that the minimum value of the k cohort-specific upper bounds would underestimate the true upper bound in the super-population, resulting in overly narrow pooled bounds.

1. **Sharpness of Intersection Bounds**

We were unable to find any evidence that the intersection bounds presented here are sharp. If we considered S=0 to be members of the population of interest that were not included in any study population, and the population of interest was a finite quantity, then it is possible that tighter bounds could be achieved using extensions of the methods presented by Chen et al (Chan 2017) or a specific application of the methods presented by Finkelstein and Shpitser (Finkelstein and Shpitser 2020). If such the population was finite, it would also be possible to generate bounds on average causal effects without assuming homogeneity of effects across study populations using these same methods (Chan 2017, Finkelstein and Shpitser 2020). However, for many questions of interest, the population of interest is potentially infinite. In the example presented here, one could consider the population of interest to be all European women who become pregnant, from the beginning of study recruitment in the earliest study and extending into the future.

1. **Description of the Avon Longitudinal Study of Parents and Children (ALSPAC)**

ALSPAC is a longitudinal birth cohort study, which aimed to recruit all pregnant women in the former Avon county, United Kingdom with a due date between April 1, 1991 and December 31, 1992, and continues to follow the offspring today. 75.3% of contacted women agreed to participate, resulting in a total of 14,541 pregnancies enrolled during the initial recruitment period. When the oldest children were approximately 7 years old, the study attempted to recruit additional eligible children who had not previously participated. The study now contains data on the offspring of 15,454 pregnancies. Further detail on the recruitment and characteristics of ALSPAC participants are available elsewhere (Boyd, Golding et al. 2013, Fraser, Macdonald-Wallis et al. 2013, Northstone, Lewcock et al. 2019). The study website contains details on available data through a fully searchable data dictionary and variable search tool (http://www.bristol.ac.uk/alspac/researchers/our-data/). Informed consent for the use of data collected via questionnaires and clinics was obtained from participants following the recommendations of the ALSPAC Ethics and Law Committee at the time. Ethical approval was obtained from the ALSPAC Ethics and Law Committee and the Local Research Ethics Committees. The previous study restricted all analyses to singleton mother-child pairs with complete data on maternal genetics, maternal pregnancy drinking behavior, and offspring outcomes, resulting in a total analytic sample of 4,457 mother-child pairs.

1. **Description of the Norwegian Mother, Father, and Child Study (MoBa)**

MoBa is a population-based pregnancy cohort study conducted by the Norwegian Institute of Public Health. Pregnant women in Norway scheduling an ultrasound between weeks 17-18 of pregnancy across Norway between 1999 and 2008 received a postal invitation to participate in the study. Women consented to participate in 41% of recruited pregnancies, and the study now includes 114,500 children, 95,200 mothers, and 75,200 fathers. Further details on the recruitment and characteristics of the cohort are available elsewhere (Paltiel, Anita et al. 2014, Magnus, Birke et al. 2016). The previous study also restricted the sample to singleton mother-child pairs with complete data on maternal genetics, maternal pregnancy drinking behavior, and offspring outcomes, resulting in a final analytic sample of 6,216 mother-child pairs.

1. **Expression for Richardson-Robins bounds**

Richardson and Robins considered a model where A and Y are binary, and the proposed instrument Z takes states {1,2,..,l} under 4 different possible assumptions:

$Z \coprod Y^{a_{1}}, Y^{a_{0}}, A^{z_{1}}, \ldots, A^{z_{l}}$

$Z \coprod Y^{a_{0}}, Y^{a_{1}}$

1. For $i\in\left\{ 1,\ldots,l \right\}, j\in\left\{ 0,1 \right\}, Z \coprod A^{z_{i}}, Y^{a_{j}}$
2. There exists a *U* such that $U \coprod Z$ and for $j\in\left\{ 0,1 \right\}, Y^{a_{j}} \coprod A, Z| U$

Under assumption (i), (ii), (iii), or (iv) for all $i,j\in\{0,1\}$, $P\left( Y^{a_{i}}=j \right)\leq g(i,j)$ where

Because $P\left( Y^{a_{0}} \right)$ and $P(Y^{a_{1}})$are variation independent, the average causal effect of A on Y, denoted $ACE\left( A\to Y \right)$ is bounded by

$$1-g\left( 1,0 \right), -g\left( 0,1 \right)\leq ACE\left( A\to Y \right)\leq g\left( 0,0 \right)+g\left( 1,1 \right)-1$$

In the previous study (Diemer, Havdahl et al. in preparation), the authors note that when multiple categorical variables are assumed to be instruments, any combination of the proposed instruments can be combined into a single joint instrument *Z* which takes states {1,2…,*l*}, where each state corresponds to a unique combination of values of the proposed joint instruments in the selected subset. The Richardson-Robins bounds could therefore be applied to any joint instrument *Z* as long as the MR conditions held both individually and jointly for each proposed instrument included in *Z*. The authors of the previous study therefore computed bounds on the average causal effect of *A* on *Y* for every combination of the 11 SNPs proposed as instruments that did not violate the instrumental inequalities.

1. **Inverse probability weighting for principal components for genetic ancestry**

Although both ALSPAC and MoBa are relatively ethnically homogenous, the authors of the previous study also conducted sensitivity analyses to evaluate the impact of residual population stratification, computing bounds in a sample inverse probability of treatment weighted for 10 principal components of genetic ancestry (Robins, Hernan et al. 2000). The authors estimated unstabilized inverse probability weights for each proposed joint instrument *Z* to account for 10 principal components as follows:

$W^{A}=$*1/*$P\left( Z \right|PC_{1},PC_{2},PC_{3},PC_{\$}, PC_{5},PC_{6},PC_{7},PC_{8},PC_{9},PC_{10})$

To estimate *W^A^*, the authors fit multinomial logistic regression models predicting *Z*, assuming the principal components contributed additively and linearly on the logit scale. Values were then back-transformed to probabilities, and the authors calculated 1/$P\left( Z \right|PC_{1},PC_{2},PC_{3},PC_{4},PC_{5},PC_{6},PC_{7},PC_{8},PC_{9},PC_{10})$ for each individual using these back-transformed probabilities in each cohort (Diemer, Havdahl et al. in preparation). In the current study, we computed intersection bounds for each combination of SNPs that did not violate the instrumental inequalities separately for these inverse probability weighted analyses, and compared them to the intersection bounds computed without inverse probability weights.

1. **R code for generating and graphing intersection bounds**

#load necessary packages
library(tidyverse)
library(cowplot)

#load alspac bounds & generate naming string
load("./alspac_anyalc.rdata")
load("./alspac_modalc.rdata")
load("./alspac_anyalc_pcs.rdata")
load("./alspac_modalc_pcs.rdata")
load("./moba_anyalc.rdata")
load("./moba_modalc.rdata")
load("./moba_anyalc_pcs.rdata")
load("./moba_modalc_pcs.rdata")

#merge datasets
#any alcohol
alspaccols <- c("instru_alspac", "ineq_alspac", "LB_alspac", "UB_alspac",

"est_alspac", "lci_alspac", "uci_alspac", "snp")

mobacols <- c("instru_moba", "ineq_moba", "LB_moba", "UB_moba", "est_moba",
 "lci_moba", "uci_moba", "snp")

colnames(alspac_anyalc_bounds) <- alspaccols
colnames(moba_anyalc) <- mobacols

anyalc_both <- merge(alspac_anyalc_bounds, moba_anyalc, by="snp")

#moderate alcohol
colnames(alspac_modalc_bounds) <- alspaccols
colnames(moba_modalc) <- mobacols

modalc_both <- merge(alspac_modalc_bounds, moba_modalc, by="snp")

#any alcohol, pcs
colnames(alspac_anyalc_pc) <- alspaccols
colnames(moba_anyalc_pcs) <- mobacols

anyalc_pcs_both <- merge(alspac_anyalc_pc, moba_anyalc_pcs, by="snp")

#moderate alcohol, pcs
colnames(alspac_modalc_pc) <- alspaccols
colnames(moba_modalc_pcs) <- mobacols

modalc_pcs_both <- merge(alspac_modalc_pc, moba_modalc_pcs, by="snp")

##generate intersection bounds for each one
intersection <- function(data){
 data <- data %>% rowwise %>% mutate(LB_inter = max(LB_alspac, LB_moba))
 data <- data %>% rowwise %>% mutate(UB_inter = min(UB_alspac, UB_moba))
}

datasets <- list(anyalc_both, modalc_both, anyalc_pcs_both, modalc_pcs_both)
data <- lapply(datasets, intersection)

## Plot results

plotbds <- function(data){
 filteredres <- data %>% filter(snp != "All Marginally Valid" &
 snp != "At Least 1 Marginally Valid" &
 snp != "At Least 2 Jointly Valid") %>%
 filter(LB_alspac<UB_alspac) %>%
 filter(LB_moba < UB_moba)
 ##rearrange so in right order again
 filteredres <- filteredres %>% arrange(-row_number())

 ##rename stuff
 filteredres$plotlab <- sapply(filteredres$instru_alspac, function(x){
 ifelse(length(x)>1,
 paste0("{",paste0(x, collapse=", "),"}", collapse=""),
 paste0(x, collapse=", "))
 })

 filteredres$plotlab <- str_remove_all(filteredres$plotlab,
 "\\_[:alpha:][:alpha:][:alpha:]")
 filteredres$plotlab <- str_remove_all(filteredres$plotlab, "\\_[:alpha:]")

 ##create ggplot
 plot <- ggplot(data = filteredres) + geom_blank() +

geom_segment(aes(x = LB_inter, xend = UB_inter, y = plotlab,

yend = plotlab), size = 1) +
 geom_vline(xintercept = 0) + scale_x_continuous(limits = c(-1.0, 1.0)) +
 labs(x = "Bounds on the average causal effect",
 y = "proposed instrument(s)") +
 theme(axis.text = element_text(size = 9, face = "bold"),
 axis.title = element_text(size = 9, face = "bold"))

 return(plot)
}

plot_list <- lapply(data, plotbds)


plot_all <- plot_grid(plot_list[[1]], plot_list[[2]], plot_list[[3]],
 plot_list[[4]], labels = c("A", "B", "C", "D"),
 label_size = 12, nrow = 2)


ggsave(filename = "pooledbounds_20220621.png", plot = plot_all,

device = "png", width = 9, height = 5.25, unit = "in")

Works Cited

Boyd, A., J. Golding, J. Macleod, D. A. Lawlor, A. Fraser, J. Henderson, L. Molloy, A. Ness, S. Ring and G. Davey Smith (2013). "Cohort profile: the ‘children of the 90s’—the index offspring of the Avon Longitudinal Study of Parents and Children." International journal of epidemiology **42**(1): 111-127.

Chan, W. (2017). "Partially identified treatment effects for generalizability." Journal of Research on Educational Effectiveness **10**(3): 646-669.

Diemer, E. W., A. Havdahl, M. R. Munafo, H. Tiemeier and S. A. Swanson (in preparation). "Bounding the average causal effect in a Mendelian randomization study with multiple proposed instruments."

Finkelstein, N. and I. Shpitser (2020). Deriving Bounds and Inequality Constraints Using Logical Relations Among Counterfactuals. Conference on Uncertainty in Artificial Intelligence, PMLR.

Fraser, A., C. Macdonald-Wallis, K. Tilling, A. Boyd, J. Golding, G. Davey Smith, J. Henderson, J. Macleod, L. Molloy and A. Ness (2013). "Cohort profile: the Avon Longitudinal Study of Parents and Children: ALSPAC mothers cohort." International journal of epidemiology **42**(1): 97-110.

Magnus, P., C. Birke, K. Vejrup, A. Haugan, E. Alsaker, A. K. Daltveit, M. Handal, M. Haugen, G. Høiseth and G. P. Knudsen (2016). "Cohort profile update: the Norwegian mother and child cohort study (MoBa)." International journal of epidemiology **45**(2): 382-388.

Northstone, K., M. Lewcock, A. Groom, A. Boyd, J. Macleod, N. Timpson and N. Wells (2019). "The Avon Longitudinal Study of Parents and Children (ALSPAC): an update on the enrolled sample of index children in 2019." Wellcome open research **4**.

Paltiel, L., H. Anita, T. Skjerden, K. Harbak, S. Bækken, S. N. Kristin, G. P. Knudsen and P. Magnus (2014). "The biobank of the Norwegian Mother and Child Cohort Study–present status." Norsk epidemiologi **24**(1-2).

Robins, J. M., M. A. Hernan and B. Brumback (2000). Marginal structural models and causal inference in epidemiology, Lww.

Swanson, S. A. (2017). "Commentary: Can we see the forest for the IVs? Mendelian randomization studies with multiple genetic variants." Epidemiology **28**(1): 43-46.
